# TP53, ATRX alterations, and low tumor mutation load feature IDH-wildtype giant cell glioblastoma despite exceptional ultra-mutated tumors

**DOI:** 10.1093/noajnl/vdz059

**Published:** 2020-01-24

**Authors:** Diana Cantero, Manuela Mollejo, Juan M Sepúlveda, Nicky D’Haene, Myriam J Gutiérrez-Guamán, Ángel Rodríguez de Lope, Concepción Fiaño, Javier S Castresana, Laetitia Lebrun, Juan A Rey, Isabelle Salmon, Bárbara Meléndez, Aurelio Hernández-Laín

**Affiliations:** 1 Department of Pathology (Neuropathology) and Instituto de Investigación i+12, Hospital Universitario 12 de Octubre, Madrid, Spain; 2 Department of Pathology, Virgen de la Salud Hospital, Toledo, Spain; 3 Department of Medical Oncology, University Hospital 12 de Octubre, Madrid, Spain; 4 Department of Pathology, Erasme Hospital, Université Libre de Bruxelles (ULB), Brussels, Belgium; 5 Department of Neurosurgery, Virgen de la Salud Hospital, Toledo, Spain; 6 Department of Pathology, Alvaro Cunqueiro Hospital, Vigo, Spain; 7 Department of Biochemistry and Genetics, University of Navarra School of Sciences, Pamplona, Spain; 8 IdiPaz Research Unit, La Paz University Hospital, Madrid, Spain

**Keywords:** ATRX, giant cell glioblastoma, next-generation sequencing, TP53, tumor mutation load

## Abstract

**Background:**

Giant cell glioblastoma (gcGBM) is a rare morphological variant of IDH-wildtype (IDHwt) GBM that occurs in young adults and have a slightly better prognosis than “classic” IDHwt GBM.

**Methods:**

We studied 36 GBMs, 14 with a histopathological diagnosis of gcGBM and 22 with a giant cell component. We analyzed the genetic profile of the most frequently mutated genes in gliomas and assessed the tumor mutation load (TML) by gene-targeted next-generation sequencing. We validated our findings using The Cancer Genome Atlas (TCGA) data.

**Results:**

p53 was altered by gene mutation or protein overexpression in all cases, while driver *IDH1*, *IDH2*, *BRAF*, or *H3F3A* mutations were infrequent or absent. Compared to IDHwt GBMs, gcGBMs had a significant higher frequency of *TP53*, *ATRX*, *RB1*, and *NF1* mutations, while lower frequency of *EGFR* amplification, *CDKN2A* deletion, and *TERT* promoter mutation. Almost all tumors had low TML values. The high TML observed in only 2 tumors was consistent with *POLE* and *MSH2* mutations. In the histopathological review of TCGA IDHwt, *TP53*-mutant tumors identified giant cells in 37% of the cases. Considering our series and that of the TCGA, patients with *TP53*-mutant gcGBMs had better overall survival than those with *TP53wt* GBMs (log-rank test, *P* < .002).

**Conclusions:**

gcGBMs have molecular features that contrast to “classic” IDHwt GBMs: unusually frequent *ATRX* mutations and few *EGFR* amplifications and *CDKN2A* deletions, especially in tumors with a high number of giant cells. TML is frequently low, although exceptional high TML suggests a potential for immune checkpoint therapy in some cases, which may be relevant for personalized medicine.

**Importance of the Study**

In the 2016 WHO classification of CNS tumors, giant cell glioblastoma (gcGBM) is a rare morphological variant of IDHwt GBM, without any particular alteration defining it as an entity. The proportion of giant cells in the tumor is not well defined to establish a final diagnosis of gcGBM, and sometimes it is only reported in the microscopic description. Therefore, the relevance of the gcGBM diagnosis is unclear. Here we study the molecular alterations and, for the first time, the tumor mutation load (TML) in one of the largest series of gcGBMs analyzed up to date. Mutation of IDH is rare, but a shift in the frequency of altered genes is observed when compared to IDHwt GBMs. Patients with gcGBMs IDHwt, *TP53*mut had better overall survival than those having GBMs *TP53*wt tumors. Our results were validated in the TCGA dataset. Most of the gcGBMs have low TML. High TML in *POLE*-mutated gcGBM suggests the potential for immune checkpoint inhibitor therapy.

**Key Points**

• IDHwt gcGBMs are *TP53*mut with unusually frequent *ATRX* mutations and few *EGFR *amplifications and *CDKN2A* deletions.
• TP53 alteration could be a driver event in gcGBM.
• Most gcGBMs had low TML. Immune checkpoint therapy could be potentially used in exceptional high TML cases.

Glioblastoma (GBM) WHO grade IV is the most frequent and malignant primary tumor of the Central Nervous System (CNS) associated with significant morbidity, mortality, and treatment resistance.^1^ Under the 2016 World Health Organization Classification of Tumors of the Central Nervous System, the 3 recognized morphological variants of IDH-wildtype (IDHwt) GBM are giant cell GBM (gcGBM), gliosarcoma, and epitheloid GBM.^2^

gcGBM occurs in adults of around 50 years old and accounts for less than 1% of all GBMs.^3^ These patients have a somewhat better prognosis than ordinary GBM, and long-term survival is found more commonly among these patients.^3–6^ However, it remains unclear whether the improved prognosis observed for gcGBM is a function of true biological differences. Histopathologically, the gcGBMs are characterized by a predominance of bizarre, multinucleated giant cells, and an occasionally abundant reticulin network. Mitosis and necrosis are frequently observed but microvascular proliferation is uncommon.^2^

In the last 2016 review of the WHO classification criteria of the CNS tumors, both morphological and molecular parameters were “integrated” for tumor classification. The molecular biomarkers for classification of adult diffuse gliomas include mutations in *IDH1/2* (IDH) and *H3F3A* (K27M mutation in diffuse midline gliomas) genes, as well as the 1p/19q codeletion (in oligodendrogliomas). In this last WHO review, gcGBM remains as a morphological variant of IDHwt GBMs, as no single genetic marker is exclusively found in gcGBM. According to previous reports, gcGBMs are characterized by frequent *TP53* (90%–70% according to different reports) and *PTEN* mutations (around 30%), while IDH mutations (5%), *EGFR* amplification (6%), and *CDKN2A* homozygous deletion (3%) are rare.^7–10^ These differences in the frequency of genetic alterations prompted some authors to place gcGBM in an intermediate position between IDHwt and IDH-mutant (IDHmut) GBM,^9^ sharing clinical and molecular characteristics with both types of tumors. In view of their particular features, these authors affirm that gcGBM clinical behavior would be closer to that of IDHmut GBM even if they rarely harbor IDH mutations.^9^

The purpose of our study is to determine whether there is a distinct molecular genetic base underlying the giant cell morphological variant of GBM.

## Materials and Methods

### Patients and Samples

We performed a retrospective study of patients diagnosed with GBM in the Hospital Universitario 12 de Octubre (Madrid, Spain), Erasme Hospital (Brussels, Belgium), and Virgen de la Salud Hospital (Toledo, Spain). The investigation was approved by the institutional review boards of each hospital. For the selection of cases, the histopathological report was reviewed. All patients with a histopathological final diagnosis of gcGBM were included. Since the exact percentage of multinucleated giant cells is not defined for the diagnosis of gcGBM in the revised WHO 2016 classification, we also selected cases where the presence of multinucleated giant cells was described in the microscopic description of the pathological report. The histological and molecular study was performed on the tumor tissue corresponding to the initial diagnosis, with the exception of 2 cases in which we did not have the initial tissue: one sample was the recurrence of a grade II astrocytoma diagnosed 22 years before and the other one was the recurrence of a GBM diagnosed 1 year before. Clinical data of the patients were obtained through a review of the patient’s charts.

DNA was extracted from selected formalin-fixed paraffin-embedded (FFPE) tumor tissues using the QIAamp DNA FFPE Tissue Kit (Qiagen) following manufacturer’s instructions and was quantified using Qubit 2.0 Fluorometer (Thermo Fisher Scientific). Hematoxylin and eosin (H&E) stained sections were examined to determine tumor areas with more than 30% of tumor cells. When there were areas of necrosis, normal parenchyma, or excessive inflammatory cells, the region to be used for DNA extraction was marked to avoid these regions and then macrodissection of the microscopically specified regions was performed by scraping the tumor section of the slides.

### Histological Evaluation

Tissue sections from all H&E-stained samples were reviewed. The following histological characteristics were recorded: percentage and size of giant cells, presence of multinucleated cells and number of nuclei, presence of lymphocytes, presence of prominent nucleoli, the existence of gemistocytic, epitheloid and primitive neuroectodermal tumor (PNET)-like foci, and presence of necrosis and vascular endothelial proliferation. The percentage of multinucleated giant cells, their average diameter, and the average number of nuclei contained were manually quantified by counting at least 1000 neoplastic cells in 10–20 random fields at 200× magnification. All cases were reevaluated histologically by a neuropathologist (A.H.L.) blinded to the final histological diagnosis and to molecular parameters.

### Next-Generation Sequencing

We used a custom Ampliseq (PCR-based) gene-targeted next-generation sequencing (NGS) panel that analyzes 30 genes that were previously demonstrated to be frequently mutated in gliomas.^11^ A second custom Ampliseq gene-targeted NGS panel used for routine glioma diagnosis was also applied to the samples in order to complete and validate the findings (Supplementary Table 1). This panel allowed to reanalyze some of the genes and added the information on hotspot mutations of the *TERT* promoter, together with the 1p/19q codeletion, *EGFR*, *EGFRviii*, and *PDGFRA* amplifications, as well as *CDKN2A* and *PTEN* homozygous deletions using a BELAC ISO 15189 accredited protocol in our laboratory.^12^ An additional accredited custom Ampliseq panel allowing hotspot *POLE* testing was applied to one case (further information on request). Libraries were constructed from 10 ng of DNA using the Ion AmpliSeq Library Kit v2.0 (Thermo Fisher Scientific), according to the manufacturer’s instructions. Libraries were multiplexed, submitted to emulsion PCR, and loaded into the chip using the Ion Chef System and sequenced using Ion Personal Genome Machine or Ion GeneStudio S5 system (Thermo Fisher Scientific), according to the manufacturer’s instructions. Average base coverage obtained and mean read length for the 30-gene and diagnostic NGS panels were 1109× and 116 bp and 2440× and 116 bp, respectively.

### Computational Analysis of NGS Data

Torrent Mapping Alignment Program (Torrent Suite v.4.2, Thermo Fisher Scientific) was used to perform the computational analyses, including sequence alignment against the hg19 reference, variant calling, and variant analysis. Variants were filtered to exclude those supported by less than 30 reads, those with an allelic frequency lower than 5%, variants outside exonic regions, and synonymous ones. Since constitutional DNA was not available to deduce germline polymorphisms, a stringent mutation detection criterion was applied in order to identify somatically acquired mutation. Mutations present in the population with a minor allele frequency greater than 1% according to the 1000 Genomes project (dbSNP build id 138) were removed. Manually curated information on variants was obtained from ExAC, COSMIC, and CbioPortal databases, as well as MutationTaster and Varsome tools to exclude polymorphisms or nonpathogenic variants. Each of the remaining variants was visualized using the Integrative Genomic Viewer (Broad Institute) and were manually selected to exclude systematic sequencing errors.^13^ Ion reporter software (Thermo Fisher Scientific) was used for copy number variation (CNV) detection, including high-level amplification of *EGFR* and *PDGFRA*, homozygous deletion of *CDKN2A* and *PTEN*, and the 1p/19q codeletion, as reported previously.^11,12^ Briefly, prediction of ploidy state is performed by using normalized read coverage across amplicons. In addition, sample read coverage is compared to a baseline coverage that is constructed from 10 male control diploid DNA samples. CNV data were filtered to exclude regions with low confidence, as recommended by the manufacturer (Thermo Fisher Scientific).

### Tumor Mutation Load Assay

Tumor mutation load (TML) was determined in 25 cases by using the Oncomine Tumor Mutation Load Assay (Thermo Fisher Scientific). This panel covers 1.7 megabase (Mb) of 409 genes involved in cancer (~1.2 Mb located in exonic regions). For this assay, 20 ng of input DNA isolated from FFPE tumor tissue were used for library preparation with Ion Ampliseq technology, following the manufacturer’s instructions. Sequencing was performed on Ion 540 Chips using the Ion GeneStudio S5 system (Thermo Fisher Scientific). Reads were aligned to hg19 using Torrent Suite 5.10 and BAM files were uploaded to Ion Reporter v5.12 for TML calculation and variant calling analysis as previously reported.^14^ Due to some samples showed FFPE deamination artifacts, we added a stricter criterion to the standard reported workflow analysis, which removed all variants with less than 30 mutant allele reads, similarly to the criterion applied for variant detection in the other panels.

### Immunohistochemistry

Immunohistochemical staining was performed on FFPE tissue sections with the following antibodies: a mouse monoclonal anti-IDH1 R132H (DIA H09; dilution 1:200; Dianova GmbH), a mouse monoclonal anti-p53 (DO-7; dilution 1:25; Novocastra Laboratories), and a polyclonal rabbit anti-ATRX (HPA001906; dilution 1:150, Sigma-Aldrich). For immunostaining, the automated instrument Leica Bond-III System was used (Leica Biosystems). Briefly, tissue sections were subsequently dewaxed, rehydrated, and subjected to antigen retrieval in a citrate-buffered saline solution (IDH1 R132H: pH 9.0 for 10 min; p53: pH 6.0 for 30 min; ATRX: pH 9.0 for 30 min). Subsequent primary antibody incubation and detection using standard avidin–biotin peroxidase methods was performed following manufacturer’s instructions and as reported.^15^

Protein p53 reactivity was scored as positive when strong nuclear positivity was present in more than 10% tumor cells.^16,17^ For ATRX immunohistochemistry, endothelial cells, cortical neurons, and infiltrating inflammatory cells were evaluated as internal positive controls. For the histological assessment of ATRX, it was considered “negative staining” only in those cases in which a loss of nuclear staining was confined to tumor cells and there was an appropriate internal positive control of adjacent nontumor cells.^15^

### Sequencing Validation

Telomerase reverse transcriptase gene promoter (*pTERT*) mutations were detected by pyrosequencing using the Pyromark Q24 ID instrument (Qiagen) as previously described.^11^ Hotspot mutations of *IDH1* and *BRAF* genes were validated by direct Sanger sequencing in an ABI PRISM 310 DNA Analyzer (Thermo Fisher Scientific)^18^ or by pyrosequencing assays.^19^

### *MGMT* Methylation-Specific PCR

For *O*^6^-methylguanine-DNA-methyltransferase (*MGMT*) promoter methylation analysis, DNA was bisulfite-treated using the EZ DNA Methylation Kit (Zymo Research), according to the manufacturer’s instructions. *MGMT* promoter methylation status was evaluated by means of methylation-specific PCR as previously reported.^20^

### Statistical Studies

Comparisons between groups were performed by two-sided Fisher exact tests, using GraphPad Statistics (http://www.graphpad.com). Statistical significance was concluded for values of *P* < .05. Kaplan–Meier survival curves and log-rank tests were used to estimate the survival distribution and were generated with SPSS (Statistical Package for the Social Sciences) v20.0 software.

### Validation Using External Databases

In order to validate and compare our results with the results obtained in a larger series of gliomas, we search in The Cancer Genome Atlas (TCGA) databases for GBMs (http://www.cbioportal.org/). In particular, we obtained data from the series of “Glioblastoma Multiforme (TCGA PanCancer Atlas).” ^21^ We performed a histopathological review of the slides of the selected cases of the TCGA databases. Cases, where the image from the frozen section was the only one available, were excluded from further analyses. Cases of the TCGA database with *IDH* hotspot mutations were excluded from all analyses. Therefore, a total of 254 patients with IDHwt tumors were used for the analyses. Details on selection of the cases are provided in Supplementary Figure 1.

## Results

### Clinical and Histopathological Features

A total of 36 GBM patients were selected: 14 with histopathological final diagnosis of gcGBMs and 22 with presence of multinucleated giant cells described in the microscopic description of the pathological report. Only one GBM had a previous low-grade biopsy, which would correspond to the so-called secondary GBM (note that this term was abandoned in the updated 2016 WHO classification as it is clinically defined). This case was excluded from further analyses as it does not conform to the current WHO classification (it harbored *IDH1* R132H mutation). The mean and median age at diagnosis of the patients were 50 and 53 years, respectively (range, 8–71), with only 3 of them younger than 20 years. A 2:1 male:female ratio was noted (23 patients were male and 12 female). The most frequent tumor locations were the temporal (46%, 16/35) and frontal (31%, 11/35) lobes. Clinical follow-up was available in all but 2 patients. Mean and median overall survival (OS) for those cases with available follow-up were 21 and 15 months, respectively. Adjuvant therapy regimen information was available for 26 patients, all of which received standard treatments. Most of them (22 patients) received radio and chemotherapy, while 3 patients received only radiotherapy. Thirteen patients received second-line treatments after recurrence.

**Figure 1.**
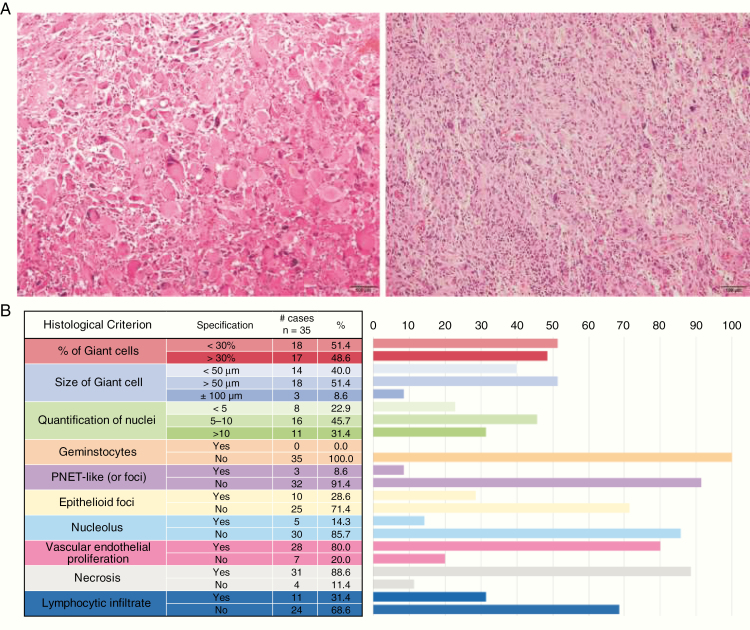
Histological evaluation of the tumors. (A) Photomicrographs of tumor tissue sections stained with hematoxylin and eosin showing multinucleated giant cell morphology. Left, a giant cell glioblastoma (gcGBM) tumor classified as having more than 30% giant cells. Right, a GBM tumor containing less than 30% giant cells. (B) Summary of the results obtained after histological reevaluation of the cases.

Half of the selected cases in this series presented more than 30% of multinucleated cells (49%, 17/35). Most of the cases presented a cell average diameter higher than 50 microns (60%, 21/35) and an average of more than 5 nuclei (77%, 27/35). Among the cases with more than 30% of giant cells, 76% (13/17) showed also a high cell size (higher than 50 microns). Concerning the rest of the histological parameters reviewed, gemistocytic cells were not observed in any case, 29% (10/35) presented small epithelioid foci, 9% (3/35) had PNET-like foci, 14% (5/35) had prominent nucleolus, and 31% (11/35) had a lymphocytic infiltrate. Almost all cases are presented with necrosis (88%, 31/35) and showed vascular endothelial proliferation (80%, 28/35) (Figure 1).

Regarding the immunohistochemical study, only one recurrent gcGBM from a low-grade glioma (case #35) was positive for IDH1 p.R132H immunohistochemistry. Positive immunohistochemical staining for p53 was detected in 89% of the cases (31/35) and loss of ATRX nuclear staining in tumor cells was observed in 24% of the cases (8/34; one case was considered noninformative since there was not an appropriate internal positive control of adjacent nontumor cells).

### Molecular Alterations in gcGBM

**Figure 2.**
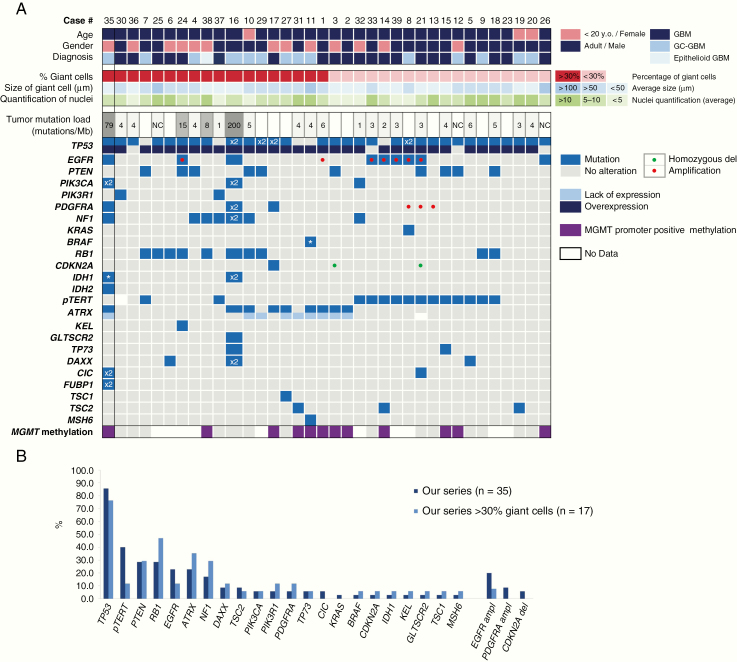
Genetic findings. (A) Summary of clinical, histopathological, and molecular findings. No genetic alterations were found in *H3F3A*, *HIST1H3B*, *CDKN2B*, *LZTR1*, MET, *FOXR2*, and *QKI*. *×*2, two mutations; *hotspot; *del*, deletion; *NC*, non contributive. (B) Frequencies of glioma gene alterations in the whole series (dark blue) and in those cases with more than 30% of multinucleated giant cells (light blue).

Genomic sequence and CNV analyses revealed at least one nonsynonymous alteration or CNV in all cases (Figure 2, Supplementary Table 2). In the whole series, only one case (#35) presented the hotspot *IDH1* p.R132H mutation. This case does not conform to the current WHO classification and was excluded from the analyses. The *TP53* gene was the most frequently mutated gene in this IDHwt series, with a total of 30 mutated cases (86%, 30/35). Immunohistochemical alteration for p53 was detected in 31 out of 35 cases (89%), with *TP53* genomic alteration identified in all except 5 of the immunohistochemically positive cases. In total, combining the immunohistochemical and NGS studies, p53 was altered in all cases (100%).

We detected alterations in the mechanism of telomere maintenance in 24 cases (69%, 24/35), either through ATRX alteration, loss of nuclear expression, and/or gene mutation (10/35, 29%) or through mutation of the promoter of *TERT* (14/35, 40%), with mutual exclusivity of both markers (Figure 2). To note, most of ATRX altered tumors had more than 30% of giant cells (80%, 8/10), while all except 2 of the *pTERT*-mutated tumors had less than 30% of giant cells (14%, 2/14). For ATRX, 8 cases lacked nuclear ATRX expression analyzed by immunohistochemistry, and mutations in the *ATRX* gene were identified in 6 of these tumors. On the other hand, 2 additional cases harbored missense gene mutations, but the nuclear expression of ATRX protein was present. Remarkably, all of these ATRX-altered tumors occurred in the absence of driver IDH and *H3F3A* hotspot mutations.

Other relevant alterations in this cohort were identified in *PTEN*, *EGFR*, *RB1*, and *NF1* and genes, which were altered in 29% (10 tumors), 26% (9 tumors mutated or amplified), 29% (10 tumors), and 17% (6 tumors), respectively. In the whole series, one case presented the hotspot *IDH1* p.R132H mutation, while another case presented *IDH1* mutations different than the hotspot (p.R49C and p.P147T, Supplementary Table 2). One single case showed the V600E mutation of *BRAF* gene. After this result, another histological review of the case was performed without finding any histological characteristics of epitheloid-GBM or pleomorphic xanthoastrocytoma or other different histological diagnoses. Because it was a case with a large presence of giant cells and besides the *BRAF* mutation it also had *ATRX* mutation, this case was not ruled out for subsequent analyses. Three cases showed *TSC2* missense mutations and an additional one truncating *TSC1* mutation, but a diagnosis of subependymal giant cell astrocytoma was discarded in these cases. Mutations of *H3F3A* were not identified in this series.

Attending to *MGMT* promoter methylation, 44% (11/25) of the samples that could be analyzed showed methylation at the *MGMT* promoter (Figure 2). No clear association of *MGMT* promoter methylation with cases with a higher percentage of giant cells was noted in this series.

### TML in gcGBM

TML could be calculated for 23 of the 25 tested cases (2 cases showed low-quality values with mean depth lower than 100× and were excluded). The mean depth for the 23 samples was 706.8× and average uniformity was 93.9%. Deamination artifacts were present in 8 of the samples, but after strict filtering of variants, the FFPE deamination estimation was dramatically reduced in all except one sample, which was therefore excluded. Most of the samples (82%, 18/22 samples) showed very low TML values (mean TML of 4.2 mutations/Mb), 2 tumors showed very high TML values (199.6 and 78.9 mutations/Mb) and 2 showed intermediate ones (14.6 and 8.3 mutations/Mb).

The mutation signatures obtained for the 2 tumors with high TML showed different patterns of somatic mutations (Supplementary Figure 2). The mutation profile of the tumor with the highest TML value (case 16) had a high proportion of C > T, C > A, T > G, and T > C changes, but low proportion C > G and T > A transversions, a mutational profile closely resembling that of other *POLE*-mutated tumors. We carried out the analysis of this sample by using a gene-targeted NGS panel validated in our laboratory for testing *POLE* mutations and identified the hotspot *POLE* p.P286R mutation in this tumor at 44% of allelic frequency. The other case (#35) was the recurrence of an *IDH1* p.R132H grade II astrocytoma. Its mutation signature was closer to the one identified in melanoma tumors and after the review of the annotated somatic nonsynonymous variants, a mutation of *MSH2* gene (p.Q681*) was identified. In contrast, the mutational profile of those cases with intermediate TML values were consistent with the spontaneous deamination of the 5-methylcytosine that was described in colorectal tumors.^14^

### Correlation of the Clinical, Pathological, and Molecular Features

The histological characteristics of the tumors were evaluated together with the NGS and the immunohistochemical results. In the series studied here, we established a percentage of delimitation of more than 30% of multinucleated cells. Excluding 2 hypermutated cases (cases 16 and 35), the average of mutations per case is approximately 4 and no significant differences between cases with or without more than 30% of giant cells were observed. However, half of the cases with more than 30% of giant cells presented ATRX alterations (53%, 9/17), while only 3 showed *pTERT* mutations (19%, 3/16 as one was not conclusive). In addition, *EGFR* amplification affected only 12% of the cases (2/17) with more than 30% of giant cells. In contrast, among the 18 cases with low content of giant cells in our series, 67% (12/18) showed *pTERT* mutation, 28% (5/18) carried *EGFR* amplification, and 11% (2/18) had ATRX alteration.

Regarding the rest of histopathological parameters registered, such as the presence of lymphocytes, prominent nucleolus, gemistocytic, epitheloid, or PNET-like morphology, necrosis, and vascular endothelial proliferation, we did not observe any correlation with the presence of particular molecular alterations. There was no relationship between the location of the tumor and the percentage of giant cells present.

### Validation of the Results Using the TCGA Data

**Figure 3.**
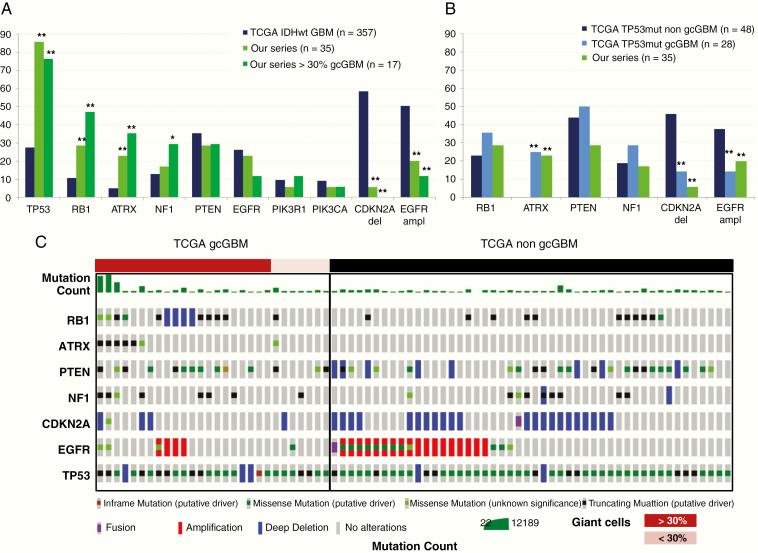
Analyses of the TCGA IDHwt GBM dataset. (A) Comparison of the gene frequency alteration between the TCGA database and our series, including that restricted to the gcGBM with more than 30% of giant cells. Note that according to the TCGA database, “Deep Deletion” indicates a deep loss, possibly a homozygous deletion. *del*, homozygous deletion in our data or deep deletion in TCGA database; *ampl*, amplification. (B) Comparison of the gene frequency alteration between *TP53*mut tumors without giant cells of the TCGA database (TCGA *TP53*mut non-gcGBBM) and those with giant cells of the TCGA database (TCGA *TP53*mut gcGBBM) and with our series. Fisher test, **P < .0001, *P < .05. (C) Oncoprint summary graph of 76 morphologically evaluated IDHwt, *TP53*mut GBM cases from the TCGA cohort. Shown are the most relevant genes and the histological review performed. Twenty-one cases showed more than 30% giant cells (dark red bar), 7 cases had less than 30% giant cells (pink), and 48 cases did not have giant cells (black).

We used the TCGA data in order to validate the results. First, we compared our data with those of the primary IDHwt GBMs reported by the TCGA. Compared to IDHwt GBMs, the mutation frequencies of *TP53*, *ATRX*, *RB1*, and *NF1* mutations were significantly higher in the gcGBMs analyzed here or in the subset of 17 tumors with higher content of giant cells (>30%) while that of *EGFR* amplification and *CDKN2A* deletion were significantly lower, and it did not change for mutations of *EGFR*, *PTEN*, or the genes coding for the PI3K subunits (*PIK3CA* and *PIK3R1*) (Fisher test, *P* > .05) (Figure 3, Supplementary Table 3).

Second, due to the TCGA data that does not specify the different morphological variants of GBM, including the gcGBM, we applied an alternative approach in order to evaluate whether we could recapitulate the findings observed in our cohort of gcGBM. Based on the fact that most of our gcGBMs showed *TP53* mutations, we selected for those IDHwt GBMs of the TCGA database that had *TP53* alterations (mutation or deep deletion, according to the TCGA) (named here TCGA *TP53*mut, 103 samples) (see Supplementary Figure 1 for case selection). The pathological review of the available tissue images of these TCGA *TP53*mut tumors revealed the presence of giant cells in 28 out of 76 evaluated tumors that could be evaluated (37%) (named TCGA *TP53*mut gcGBM), with 21 of them showing more than 30% of giant cells (Figure 3). We observed that mutation frequencies of *RB1*, *ATRX*, and *PTEN* significantly increased in this TCGA *TP53*mut gcGBM group, while *CDKN2A* deletion and *EGFR* amplification diminished, when compared to TCGA *TP53*wt tumors (Supplementary Table 3). However, among all these genes, only the frequencies of *ATRX* mutation, deletion of *CDKN2A*, and amplification of *EGFR* showed significant differences between TCGA *TP53*mut gcGBMs and TCGA *TP53*mut non-gcGBMs, which was confirmed in our series (Figure 3). Furthermore, these differences increased in the TCGA dataset with more than 30% of giant cells, where 29% (6/21) of the cases had *ATRX* mutation, 19% (4/21) had *EGFR* amplification, and 19% (4/21) had *CDKN2A* alterations (mutation or deep deletion) (Figure 3, Supplementary Table 3).

Taking together the histological review and the genetic data of the TCGA cases, the frequencies of gene mutation observed in our series could be recapitulated in the identified 28 TCGA *TP53*mut gcGBMs. These results confirm that gcGBMs have higher frequencies of *TP53* and *ATRX* while lower *EGFR* amplification and *CDKN2A* loss compared to unselected IDHwt tumors, or to IDHwt, *TP53*mut tumors without giant cells.

To note, 2 of the TCGA gcGBMs *TP53*mut had very high mutation counts (TCGA-06-5416 and TCGA-19-5956). Due to our finding of a *POLE*-mutated case with high TML, we searched for mutations of this gene. In both cases, a hotspot pathogenic *POLE* mutation was identified (p.V411L and p.A456P, respectively).

### Analyses of OS

**Figure 4.**
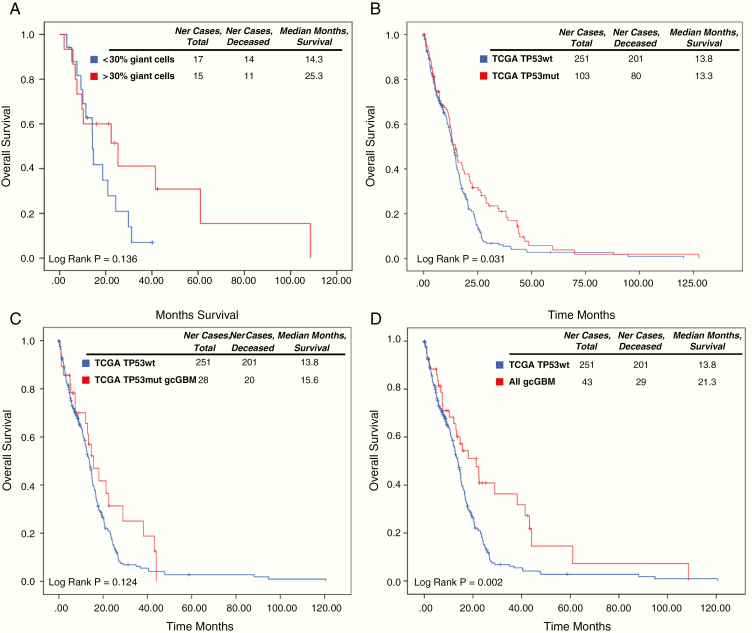
Analyses of overall survival (OS) in the cohort of gcGBM studied here and the TCGA cohort of IDHwt tumors. (A) Comparison of GBM patients of our series with more than 30% of giant cells and those with less than 30%. (B) Comparison of TCGA *TP53* and *TP53*mut patients. (C) Comparison of TCGA *TP53*wt and TCGA *TP53*mut gcGBM patients. (D) Comparison of TCGA *TP53*wt and all gcGBMs patients (our series and the TCGA cases).

In our series, Kaplan–Meier survival curves showed a tendency to greater survival in those patients with more than 30% of giant cells in the tumor versus those with less than 30%, with median OS of 25.3 and 14.3 months, respectively (log-rank test, *P* = .136) (Figure 4).

Analyses of the IDHwt TCGA data allowed us to compare the OS of *TP53*wt and *TP53*mut patients. We observed significant differences between both groups of patients (log-rank test, *P* = .031), with a median OS of *TP53*wt and *TP53*mut groups of 13.8 and 13.3 months, respectively. A nonsignificant difference was observed when TCGA *TP53*wt patients were compared to the identified TCGA *TP53*mut gcGBM patients (log-rank test, *P* = .124), although the tendency of the curves suggested a better OS of TCGA *TP53*mut gcGBM (median 15.6 months). Taking into account all gcGBM patients identified in this and the TCGA cohort (median OS of 21.3 months), significant differences were observed between them and TCGA *TP53*wt patients (log-rank test, *P* = 0.002) (Figure 4).

## Discussion

The molecular alterations that occur in the giant cell phenotype are not yet fully understood. gcGBMs have low rates of IDH mutations^9^ (only one secondary GBM IDH mutated identified in this series). As a consequence, it is currently included as a histological variant of “classic” IDHwt GBM in the last 2016 WHO classification system, even when there is some evidence that gcGBM presents different clinical characteristics (younger patients) and improved prognosis (overall and progression-free survival) over GBM.^4–6^ However, although the rarity of IDH mutation in gcGBM reinforces the current classification, it should be noted that the giant cell phenotype can also appear in IDH-mutated tumors. The lack of well-defined histological or molecular criteria makes that the presence of giant cells is sometimes even not described in the pathological reports. According to the WHO criterion, the designation of gcGBM is based on the “presence of numerous multinucleated giant cells.” However, no exact percentage is specified when describing this histological variant, and therefore a more objective definition based on the correlation between histological and molecular parameters would be needed.

Our results and those of other authors showed a high incidence of *TP53* mutations while low incidences of *EGFR* amplification and *CDKN2A* deletion.^7–10^ Furthermore, according to our study and the one of Oh et al.,^9^ gcGBMs also have a significantly higher incidence of ATRX alteration and a lower one of *TERT* promoter mutations when compared to “classic” IDHwt GBMs. Remarkably, our study shows that this difference becomes more relevant when considering a strict criterion based on the content of giant cells (>30% in our study). Here, we have validated the molecular findings obtained by performing a histopathological review of the cases from the TCGA-GBM database. We showed that in selected TCGA *TP53* mutant, IDHwt tumors, giant cells were present in at least 37% of the cases.

Our work extends the molecular characterization of gcGBM by analyzing the most frequently altered genes in gliomas. To note, we identified a significant higher incidence of *RB1* and *NF1* mutations in gcGBM (and in those with >30% giant cells) compared to IDHwt or IDHwt–*TP53*wt GBMs, which were not reported previously. According to the TCGA database, *RB1* mutation correlates with *TP53* mutation in IDHwt GBMs, which may suggest that this alteration is not exclusive of gcGBMs but of *TP53*-mutated tumors. Comparison of gene alteration frequencies within TCGA *TP53*mut cases between tumors containing or not giant cells did not reveal significant differences for *RB1*, *NF1*, or *EGFR* mutation. However, *ATRX* mutation, *EGFR* amplification, and *CDKN2A* deletion were significantly increased (*ATRX*) or decreased (*EGFR* and *CDKN2A*) in TCGA *TP53*mut gcGBMs compared to TCGA *TP53*mut non-gcGBMs tumors. Further validation would be needed to increase the number of cases supporting these data.

A *TP53* mutation is a genetic event that occurs in all types of cancer, and it is thought to be inactivated in the early stage of tumorigenesis.^22^ Furthermore, due to its function in centrosome duplication and in cell-cycle arrest, p53 has a role in genome stability.^23,24^ The high rate of *TP53* alteration in gcGBM suggests that this gene has a main role in these tumors and that it could represent one of the earliest events in the gliomagenesis of gcGBMs. Further, it may point out molecular differences at the initial steps of the tumor from that of the “classic” GBMs, and also from IDH-mutated GBMs, through a TP53-dependent genomic instability mechanism, as was previously suggested.^7^

Neoplastic cells need to maintain their telomere length for achieving immortality. It can occur by upregulation of the telomerase (in most of the tumors) or through the alternative lengthening of telomeres (ALT), a homologous recombination-based mechanism (in 10%–15% of cancers). Mutations of *ATRX* (or lack of protein expression) have been linked to the ALT mechanism,^25^ and mutations of the promoter of *TERT* result in upregulation of the telomerase, both events being mutually exclusive in CNS tumors. In IDHwt GBMs, maintenance of the telomeres is mainly achieved through mutations of the *TERT* promoter, while in IDH-mutated (non-codeleted) gliomas, ATRX alteration is frequently observed. Interestingly, although most of the gcGBMs are IDHwt, they show an unusually frequent alteration of ATRX, particularly those with higher contents of giant cells, suggesting that these tumors could manage to maintain their telomere length by activating the ALT mechanism rather than by upregulation of the telomerase. It has been reported that ATRX deficiency, in and of itself, is not sufficient for ALT activation, and additional genetic or epigenetic changes are required such as *IDH1*, *TP53*, or *H3F3A* mutations or cell cycle checkpoint dysfunction.^26^ In the case of gcGBMs, absence of IDH and *H3F3A* mutations may suggest a role for TP53 or other yet unknown factors contributing for ALT.

It is interesting to note that most of the gcGBMs were characterized by a low TML, in accordance with the low mutation rate detected by the gene-targeted glioma panel used here and with the data reported in a recent report of 10 gcGBM tumors.^27^ Two cases, however, displayed very high TML values. One of them had a mutational signature pattern resembling that of tumors with *POLE* hotspot pathogenic mutation and a *POLE* mutation was confirmed by NGS analysis in this tumor. In fact, the 2 gcGBM cases of the TCGA database presenting exceptional mutation counts also had pathogenic mutations of *POLE* gene. Ultramutated tumors carrying somatic *POLE* mutations were reported previously^28^ in 6 GBM cases (3 of them with *MSH6* germline mutations), and they also showed the presence of multinucleated giant tumor cells. This suggests that these patients may be stratified for checkpoint blockade immunotherapy. In fact, a clinical and antitumor immune response to PD-1 blockade was described in a GBM patient with *POLE* germline mutation.^29^ Our study here suggests that the mutation load assessed through targeted gene panels could be a useful tool for the stratification of these patients. The second case with high TML in our study, which is a recurrence from a low-grade tumor, had a mutation signature resembling that of melanoma tumors, and the identified mutation in *MSH2* gene suggests that the hypermutator profile in this case could be related to adjuvant temozolomide treatment, similarly to what was described in tumors harboring *MSH6* mutation after alkylator chemotherapy.^30^

We performed immunohistochemical staining for mismatch repair (MMR) proteins (MLH1, MSH2, MSH6, and PMS2), as well as for PDL1 in these 2 cases with high TML. Both cases showed loss of MMR protein expression (case #16 for PMS2 and case #35 for MSH2 and MSH6) and negative PDL1 immunostaining (data not shown). Loss of expression of MMR proteins and/or microsatellite instability was previously reported in gcGBMs by Martinez et al.^8^ in around 30% of a total of 10 patients with gcGBM. Our results and those of Martinez et al. suggest that these markers could be used in the context of possible immunotherapy using checkpoint inhibitors. Further analyses, however, would be needed to validate these findings.

Finally, in our cohort we observed a better OS of gcGBM patients with a higher percentage of giant cells (>30%). By using external data of IDHwt tumors from the TGCA database we could confirm the better OS of *TP53*mut GBMs and *TP53*mut gcGBM patients compared to *TP53*wt tumors. Further studies, however, are needed to validate these findings.

Through the integration of the histological and genetic features of the tumors, we have demonstrated a pathological and molecular correlation in gcGBM. Cases with combined alterations in p53 and ATRX are prone to have giant cells, in higher percentages and greater in size than those tumors not carrying these mutations. According to our results, we suggest that the percentage and size of the giant cells in order to define the gcGBM variant should be defined. In our study, a good percentage of delineation was 30% and an average diameter was greater than 50 microns, because these 2 parameters had a better correlation with the molecular alterations. However, it is worth noting that all the genetic alterations identified here can also be found in other GBMs that do not have morphologically giant cells and, therefore, the differences with the “classic” IDHwt GBM are limited to a change in gene frequency alteration. To date, there is no single genetic marker that is only altered in gcGBMs and, therefore, the importance of the giant phenotype still requires a more detailed analysis.

In conclusion, our results provide more information about the histopathological, genetic, and clinical features that characterize GBM cases with giant cells. A better histological and molecular characterization can allow a closer diagnosis and consequently may be important for the potential management of these patients.

## Supplementary Material

vdz059_suppl_Suplementary_Table_1

vdz059_suppl_Suplementary_Table_2

vdz059_suppl_Suplementary_Table_3

vdz059_suppl_Suppl_Fig_1

vdz059_suppl_Suppl_Fig_2

vdz059_suppl_Supplementary_Table_Figure_Legends
