# Supplementary material for: TP53, ATRX alterations, and low tumor mutation load feature IDH-wildtype giant cell glioblastoma despite exceptional ultra-mutated tumors
Source: Neurooncol Adv. 2020 Jan 24;2(1):vdz059. doi: 10.1093/noajnl/vdz059 (PMC7212869; doi:10.1093/noajnl/vdz059)
Supplement: vdz059_suppl_Suppl_Fig_1 [file vdz059_suppl_suppl_fig_1.pptx]

## Slide 1
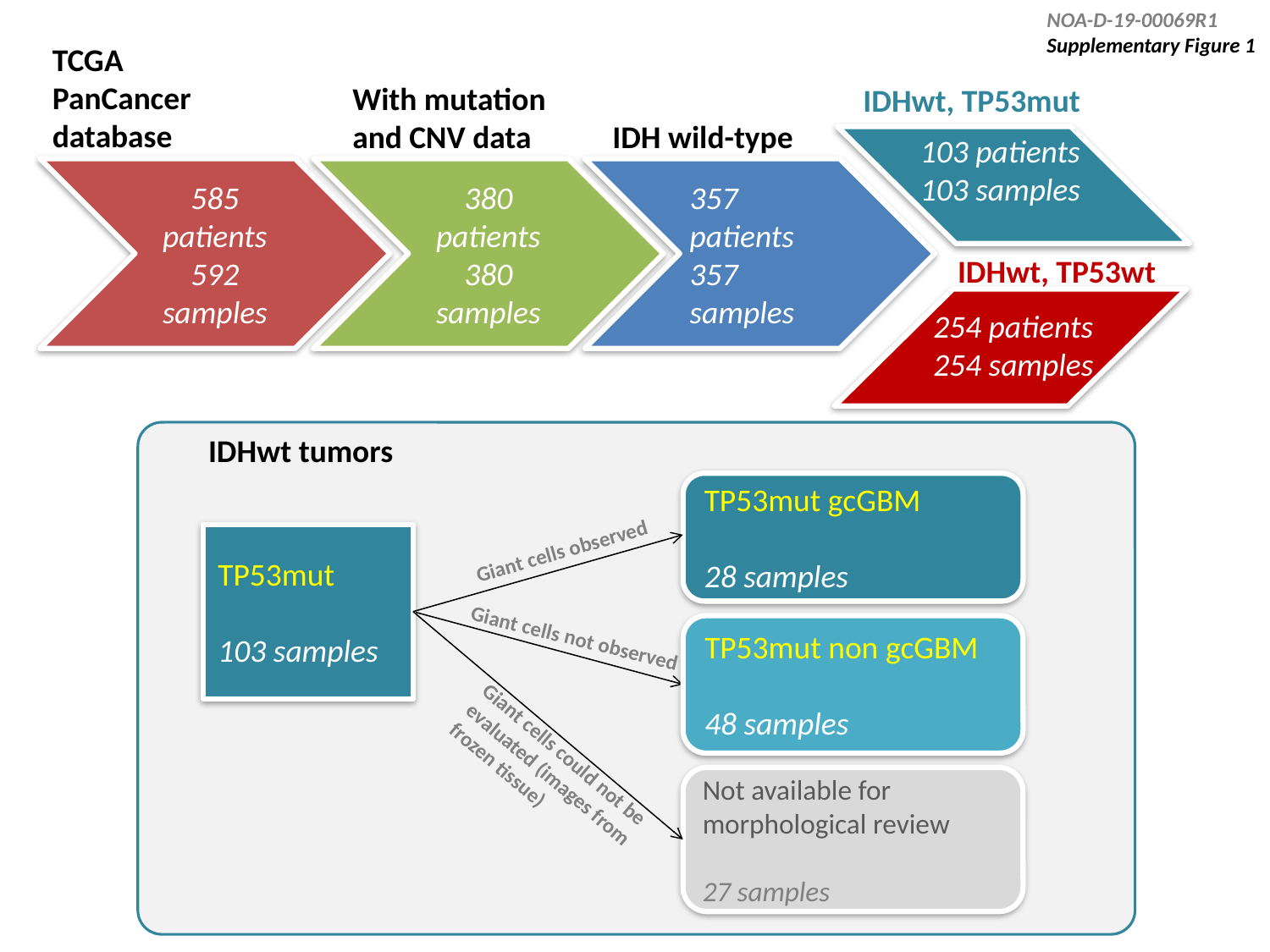

NOA-D-19-00069R1
Supplementary Figure 1
TCGA PanCancer database
With mutation and CNV data
IDHwt, TP53mut
IDH wild-type
103 patients
103 samples
585 patients
592 samples
380 patients
380 samples
357 patients
357 samples
IDHwt, TP53wt
254 patients
254 samples
IDHwt tumors
TP53mut gcGBM
28 samples
TP53mut
103 samples
Giant cells observed
TP53mut non gcGBM
48 samples
Giant cells not observed
Giant cells could not be evaluated (images from frozen tissue)
Not available for morphological review
27 samples
