# Supplementary material for: TP53, ATRX alterations, and low tumor mutation load feature IDH-wildtype giant cell glioblastoma despite exceptional ultra-mutated tumors
Source: Neurooncol Adv. 2020 Jan 24;2(1):vdz059. doi: 10.1093/noajnl/vdz059 (PMC7212869; doi:10.1093/noajnl/vdz059)
Supplement: vdz059_suppl_Suppl_Fig_2 [file vdz059_suppl_suppl_fig_2.pptx]

## Slide 1
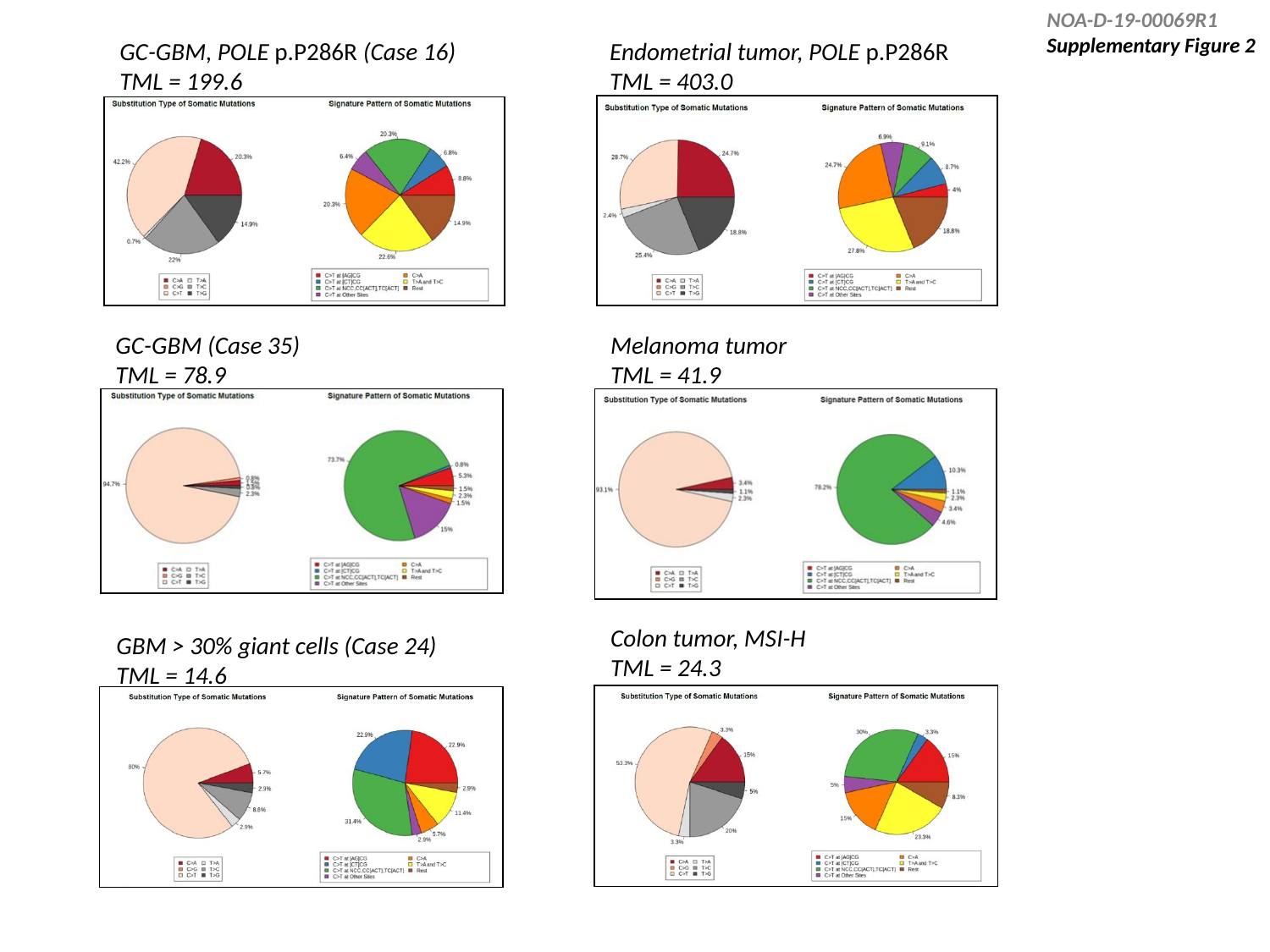

NOA-D-19-00069R1
Supplementary Figure 2
GC-GBM, POLE p.P286R (Case 16)
TML = 199.6
Endometrial tumor, POLE p.P286R
TML = 403.0
GC-GBM (Case 35)
TML = 78.9
Melanoma tumor
TML = 41.9
Colon tumor, MSI-H
TML = 24.3
GBM > 30% giant cells (Case 24)
TML = 14.6
