# Supplementary material for: TP53, ATRX alterations, and low tumor mutation load feature IDH-wildtype giant cell glioblastoma despite exceptional ultra-mutated tumors
Source: Neurooncol Adv. 2020 Jan 24;2(1):vdz059. doi: 10.1093/noajnl/vdz059 (PMC7212869; doi:10.1093/noajnl/vdz059)
Supplement: vdz059_suppl_Supplementary_Table_Figure_Legends [file vdz059_suppl_supplementary_table_figure_legends.docx]

**Supplementary Table and Figure Legends**

**Supplementary Table 1.** List of genes and copy number variations contained in the gene-targeted next-generation sequencing panels.

**Supplementary Table 2.** Variants identified in the whole GBM series analyzed here.

**Supplementary Table 3.** Frequencies of gene alteration and significant differences with the TCGA database.

**Supplementary Fig. 1.** Selection of cases among the 585 patients of the TCGA PanCancer database. IDH mutant cases were excluded. The TP53mut tumors included those with *TP53* mutation and deep deletion. According to the TCGA, “Deep Deletion” indicates a deep loss, possibly a homozygous deletion.

**Supplementary Fig. 2.** Mutational signature of tumors with high tumor mutational load compared to tumors showing the same mutational profile.
